# Percolation of temporal hierarchical mobility networks during COVID-19

**DOI:** 10.1098/rsta.2021.0116

**Published:** 2022-01-10

**Authors:** Haoyu He, Hengfang Deng, Qi Wang, Jianxi Gao

**Affiliations:** ^1^ Department of Computer Science and Center for Network Science and Technology, Rensselaer Polytechnic Institute, Troy, NY 12180, USA; ^2^ Department of Civil and Environmental Engineering, Northeastern University, Boston, MA 02115, USA

**Keywords:** percolation theory, COVID-19, human mobility, network science

## Abstract

Percolation theory is essential for understanding disease transmission patterns on the temporal mobility networks. However, the traditional approach of the percolation process can be inefficient when analysing a large-scale, dynamic network for an extended period. Not only is it time-consuming but it is also hard to identify the connected components. Recent studies demonstrate that spatial containers restrict mobility behaviour, described by a hierarchical topology of mobility networks. Here, we leverage crowd-sourced, large-scale human mobility data to construct temporal hierarchical networks composed of over 175 000 block groups in the USA. Each daily network contains mobility between block groups within a Metropolitan Statistical Area (MSA), and long-distance travels across the MSAs. We examine percolation on both levels and demonstrate the changes of network metrics and the connected components under the influence of COVID-19. The research reveals the presence of functional subunits even with high thresholds of mobility. Finally, we locate a set of recurrent critical links that divide components resulting in the separation of core MSAs. Our findings provide novel insights into understanding the dynamical community structure of mobility networks during disruptions and could contribute to more effective infectious disease control at multiple scales.

This article is part of the theme issue ‘Data science approaches to infectious disease surveillance’.

## 1. Introduction

The unprecedented pandemic of the coronavirus disease 2019 (COVID-19) is affecting more than 200 countries and infected more than 32 million causing 578 530 deaths in the USA as of 9 May 2021 [1]. In a short one-year period, the USA has experienced two waves of transmissions [2]. In response to the fast and dynamic transmission of SARS-CoV-2, different levels of government have employed restricting population movement as a key non-pharmaceutical intervention (NPI) in limiting contacts and increasing social distancing [3]. These measures have caused significant changes in people’s mobility patterns [4]. Also, the employment of stay-at-home orders has substantially reduced long-distance travel as well as local commuting [5]. Indeed, human mobility is both a key driver of and NPI to control COVID-19 [6–8].

Numerous studies have investigated the correspondence between mobility patterns and the spread of infectious disease such as SARS, influenza and malaria [9–13] as well as COVID-19 in different countries [14–20]. Studies have shown that the early spatial patterns of COVID-19 infection in China correspond to the human mobility fluxes and such correlation drops as some local control policies were executed [21,22]. Other studies support the finding that regions and places with high volumes of mobility are contributing to the spread of the deadly virus [15,23–25]. On the flip side, social distancing and mobility reduction are associated with a decreased level of transmission of COVID-19 [21,26,27].

Mobility-based networks are constructed to accurately model the transmission of COVID-19 in social networks, but they suffer from a few limitations. First, when building the networks, compromise on resolution or scale is often necessary to account for both the spatial and temporal dynamics of mobility networks. Studies have aggregated human mobility on the census block group or county levels [28–32]. The aggregation removes details and information on other levels and leads to inaccurate prediction of transmission patterns. Second, previous studies validate the effectiveness of inter-city travel restrictions in reducing the imported COVID-19 incidence rate [23,33]. Yet, there is still limited understanding of how local measures and responses could change the mobility network structure. Specifically, communities may still face challenges from intra-city mobility, which must be considered in network models. Third, a recent study has discovered the percolation process and phase transition in human mobility networks on the county level, which indicates the possibility of devising effective strategies to control mobility flows at critical bridges and contain the transmission of COVID-19 [31]. However, it is unknown if percolation processes govern the structural changes in mobility networks with a higher geographical granularity or multi-level mobility networks.

To address these limitations, here we propose a multilevel approach to capture the hierarchical and the dynamical property of mobility networks. We construct two-layer hierarchical networks of 378 Metropolitan Statistical Areas (MSAs) in the USA. Such dynamic multiplex networks consist of a macro-level and meso-level layer for daily mobility. On the macro level, the MSAs are nodes, and the travels among the metropolises are links. On the meso level, we treat census block groups as nodes and the travels between as links. This hierarchical construction allows us to analyse local and long-range trips simultaneously and examine their interactions. Thus, the multiplex network supports our effort to uncover the associations between different layers and node attributes such as COVID-19 incidence rate and collective response metrics.

To understand the structural changes of multiplex networks, we adopt an analytical approach that originated from percolation theory. As a fundamental concept in network science, percolation theories are studied extensively, contributing to understanding and conducting materials and network-based applications [34–36]. Specifically, percolation theory is an essential step for coping with complex models and dynamical processes occurring on the networks [37–44]. It is a crucial approach to identify the hierarchical structure and determine any discontinuous phase transition within the complex systems. Previous applications in network science include critical phenomena in urban traffic planning [45–47], epidemic modelling [48,49] and cascading processes on networks [50,51]. These strengths make the approach effective in better understanding the hierarchy of dynamic mobility networks and thus unearth variations in mobility stabilization and emergent structure patterns.

## 2. Mobility data

We use the Social Distancing Metrics dataset from SafeGraph to quantify the daily travel changes (see Data availability). The data track the GPS pings of more than 20 million anonymous mobile devices and records the related information of their daily movements, including travelling trajectory, home-dwelling time, distance travelled from home, etc. The smallest geological unit used is the census block group; therefore, a device’s location is spatially joined to the census block group containing it. Here the home block group of a device is defined as its common nighttime (18:00–7:00) census block group for 6 consecutive weeks. The mobility network is built based on the movements between different block groups. The dataset has a field *destination_cbgs* which is a dictionary that reports the destination census block groups and the number of devices that visited them from each home census block group. The dataset ranges from 1 January to 31 December 2020, allowing us to study human mobility patterns before and after the national emergency declared on 13 March 2020. The data track each device’s daily trajectory among census block groups in the USA during daytime (7:00–18:00). A human mobility network is formed based on it, in which a directed link from block group i to block group j is created if a device is detected living in block group i and travelling to block group j within the same day. The weight is the number of devices detected travelling from i to j.

## 3. The hierarchical networks structure of the USA

**Figure 1.**
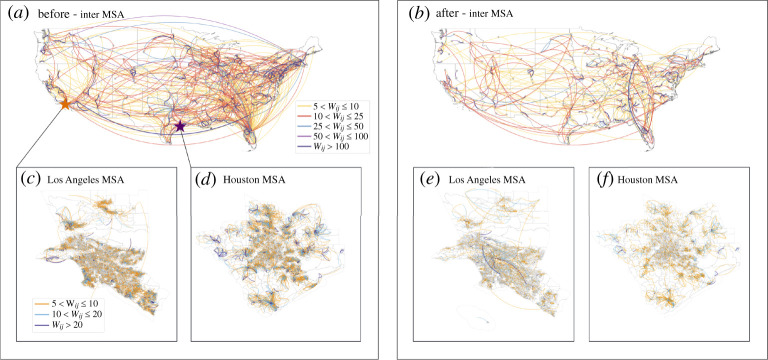
Inter- and intra-MSA mobility networks before and after the national emergency declaration. In (*a*,*b*), the nodes represent MSAs before and after the national emergency declaration. The colours of the links demonstrate the number of 7-day average flux normalized by the device count between MSAs. Panel (*c*,*d*) demonstrates the intra-MSA networks of Los Angeles MSA and Houston MSA before the national emergency declaration. The nodes are census block groups and the colours are based on the numbers of 7-day average normalized influx between pairs of nodes. (Online version in colour.)

We build a mobility-based, hierarchical network of 378 Metropolitan Statistical Areas (MSAs) in the USA (figure 1). Our approach aggregates and normalizes the regional travel flux based on the number of devices observed in both origin and destination regions on two levels: inter-MSA (figure 1*a*,*b*) and intra-MSA (figure 1*c*–*f*). For the intra-MSA networks, denoted as α-networks, we take each census block group as a node. A directed link from block group i to block group j is created if a device is detected living in block group i and travels to block group j within the same day. The weight is the number of travellers. One issue that needs to be addressed is that human mobility is significantly different between weekdays and weekends, and yet patterns from both periods are important and should be considered in this research. Because of this, we average the data from every 7 consecutive days (3 days before to 3 days after) to construct the networks. This approach ensures every network includes a set of weekdays and weekends. Thus, it captures a weekly periodicity of routine mobility and reduces the effects of outliers. The edge weight is the mean of the weights on the same edge for these 7 days.

For the inter-MSA networks, denoted as ω-networks, we aggregate all block groups in an MSA and treat it as a node. Therefore, there are 378 nodes in the inter-MSA networks. The weight of a link is the mean of the normalized number of total travellers between two MSAs for 7 consecutive days.

To simulate the mobility flow from node i to node j to calculate the probability of a infected person travelling from i to j, we let population of node i be m, infected population of i be n, and k people travel from i to j. Thus, we can roughly present the probability of infection for each person in i be n/m and we can calculate the probability of not spreading COVID-19 to j as a function of n/m, $f(n/m)=1-{\left(1-(n/m)\right)}^{k}$. Taking its binomial expansion, we have

f(nm)=1−(1−nm)k=1−∑s=0k(ks)1k−s(−nm)s=nkm−k(k−1)2(nm)2+⋯

Since the infected population at any particular time point is relatively small compared with total population of that area, we can consider n/m≪1 and terms in this binomial series are converging to 0 rapidly. Thus, the value from this function is largely determined by the first term nk/m and the rest of the terms can be ignored. Since the infected population, n, is an inaccurate constant for various reasons (i.e. false-negative rate, asymptomatic individuals), we use k/m to represent the weight of links. Therefore, we develop a normalized weighting approach using device count to mitigate the biases of mobility flow:

$$W_{ij}=\frac{R_{ij}}{D_{i}}+\frac{R_{ji}}{D_{j}},$$

where $R_{ij}$ is denoted as the number of people travelling from node i to j, and $D_{i}$ is the device count of node i on the same day. The constructed hierarchical networks simultaneously examine local mobility flows within MSAs and long-distance travel between MSAs. The construction also enables systematic exploration of the interactions between the two levels.

To show the computational advantage of the hierarchical network model compared with traditional ones, we run the percolation process on both the hierarchical model and the traditional model with the same day of data from our data set. The average time of analysing the percolation process of one-day data is about 30 min for the hierarchical model and about 10 h for the traditional model using the same computer. The processing time of the traditional model cannot support real-time mobility analysis, and thus the hierarchical model is necessary.

Figure 1 shows significant changes in mobility flows before and during COVID-19. Figure 1*a* shows the ω-network structure in the week of 1 February, and figure 1*b* in the week of 1 June. As the weight of the edge gets larger, the colours of the links in graphs get darker. Comparing figure 1*a*,*b*, we observe that edge weights of most links decrease substantially. Therefore, fewer people travel between MSAs and the entire network becomes sparser. Figure 1*c*,*d* shows the intra-MSA network (i.e. α-network) structure of Los Angeles and Houston in the week of 1 February and figure 1*e*,*f* the one in the week of 1 June. The ω- and α-networks display similar geographical distributions even though the networks become sparser due to a decline in mobility.

## 4. Intra-MSA percolation

**Figure 2.**
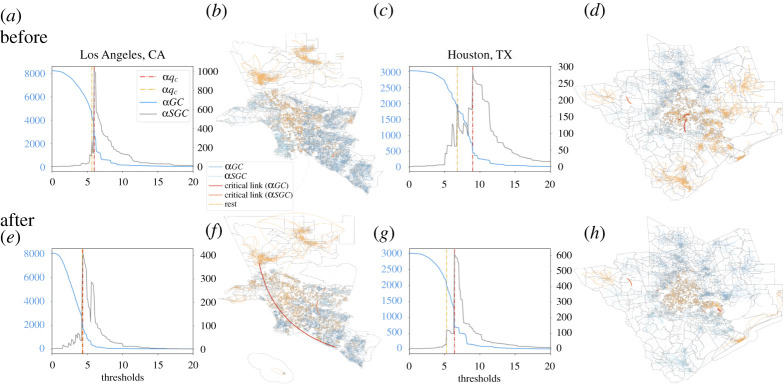
Percolation process of mobility networks in Los Angeles and Houston MSAs. Panel (*a*) shows the two largest connected components(αGC and αSGC) with the change of $\alpha q_{c}$ in the week of 29 January to 4 February 2020 in Los Angeles MSA. Panel (*b*) shows its network when the weight of edges equals $\alpha q_{c}$ in the week of 29 January to 4 February 2020. Panel (*c*) shows αGC with the change of $\alpha q_{c}$ in the week of 29 January 29 to 4 February 2020 in Houston MSA. Panel (*d*) shows the network of Houston MSA when the weight of edges equals $\alpha q_{c}$ in the week of 29 January to 4 February 2020. (*e*) shows the two largest connected components with the change of $\alpha q_{c}$ in Los Angeles MSA size in the week of 29 May to 4 June 2020. Panel (*f*) shows the network with the change of $\alpha q_{c}$ in Los Angeles MSA in the week of 29 May to 4 June 2020. Panel (*g*) shows the network with the change of $\alpha q_{c}$ in Houston MSA in the week of 29 May to 4 June 2020. Panel (*h*) shows the network map of Houston when the threshold of the weight of edges equals $\alpha q_{c}$ in the week of 29 May to 4 June 2020. (Online version in colour.)

We apply a percolation-based approach to analyse the connected components of both the α- and ω-networks and study their phase transitions. We first set the threshold of the edge weight 0 and increase it by a stepsize after each iteration. With each increase, the edges with weights lower than the threshold are removed from the graph and a new graph is generated with the remaining edges and nodes. The method is adapted from other research such as a traffic study in Beijing which removes less crowded to more crowded routes [45] and a study on the regional structure of Britain by adding edges to cities sorted by distancing between them from nearest to furthest [52]. After each update, we calculate the connected components, especially the largest, i.e. the giant, component (GC), and the second-largest, i.e. the second giant, component (SGC). Figure 2*a*,*c* shows that the size of αGC of an intra-MSA network declines and αSGC increases. At the critical point of $\alpha q_{c}$, the largest component experiences a sudden drop and αSGC simultaneously reaches its maximum size.

The observed change aligns with results reported in [31], showing that mobility networks on different scales, i.e. county and block groups, experience similar percolation processes. It also indicates that the intra-MSA α-network experiences a phase transition at $\alpha q_{c}$. After the transition, the network becomes sparser, and components are more likely to disconnect from each other.

Besides $\alpha q_{c}$, we also examine a second critical point $\alpha q_{c2}$, at which αSGC is the largest before $\alpha q_{c}$. Thus, $\alpha q_{c}$ and $\alpha q_{c2}$ indicate that the network experiences hierarchical phase transitions [31,45].

The percolation approach allows us to examine networks in different MSAs. We analyse the mobility data from 29 January to 4 February 2020 in Los Angeles MSA and find the critical point at $\alpha q_{c}=6$ (red line in figure 2*a*). At this point, the second-largest component experiences a significant increase, and its size reaches 1001 with a total of 8211 census block groups which is at its maximum. Simultaneously, about the same amount of nodes disconnects from αGC, indicating that αSGC separates from the largest one.

It is worth noting that the network becomes sparse at $\alpha q_{c}$ (red line in figure 2*a*) where the largest possible component other than αGC is disconnected from αGC. At this point, αGC experienced the most significant decrease throughout the whole process and constitutes only a small portion, 33.13% (2720 out of 8211 block groups), of the original network. Thus, the network lost most of its connectivity and cannot effectively spread COVID-19 inside the MSA. In other words, COVID-19 is more likely to spread within each subcomponent without transmitting to other ones. Another finding is that prior to $\alpha q_{c}$, we discover a less significant phase transition at $\alpha q_{c2}$ (yellow line in figure 2*a*) where the MSA can also be partitioned into subcomponents. The same patterns are observed in Houston MSA (figure 2*c*).

After identifying the critical points, i.e. $\alpha q_{c}$ and $\alpha q_{c2}$, we locate the critical links whose elimination causes αSGC to disconnect from the largest one. Figure 2*b* shows the network of Los Angeles MSA in the week of 29 January when the threshold is $\alpha q_{c}$. The dark blue component is αGC, the light blue one is αSGC, and the orange the rest. The critical link is marked in red (figure 2*b*) with the weight of $\alpha q_{c}$. The link connects αGC in the east MSA Los Angeles and αSGC in the west. Similarly, in Houston MSA (figure 2*d*), the critical link separates αGC in the North and αSGC in the South. The findings align with a previous study on the county level [31] and suggest that local mobility networks have bottlenecks when going through the percolation process.

Figure 2*e*–*h* demonstrates the same percolation process after the state emergency declaration (29 May 29 to 4 June 2020). Comparing figure 2*a*,*e*, we find that the sizes of both αGC and αSGC are similar. However, $\alpha q_{c}$ and $\alpha q_{c2}$ are smaller than those before the national emergency declaration. A similar pattern is observed in Houston MSA (see figure 2*c*,*g*). Thus MSAs are less robust after the national emergency declaration and they could be broken into isolated clusters with smaller sacrifices to effectively contain COVID-19 within a small area. At the $q_{c}$s, the locations of αGC and αSGC in Los Angeles MSA almost remain the same as before (figure 2*b*) and after (figure 2*f*). The similarity is also observed in Houston MSA (figure 2*d*,*h*). However, local mobility decreases substantially after the state emergency declaration, and thus the components become more sparse. A sparse network means that mobility networks could be disconnected and controlled more easily. Also, the weights of edges decline in a similar proportion and the patterns of major clusters at $q_{c}$ are almost the same before and after the national emergency declaration. Thus, it is feasible to devise effective measures in controlling the spread of COVID-19 locally.

## 5. Intra-MSA correlation of $\alpha q_{c}$ with other attributes

**Figure 3.**
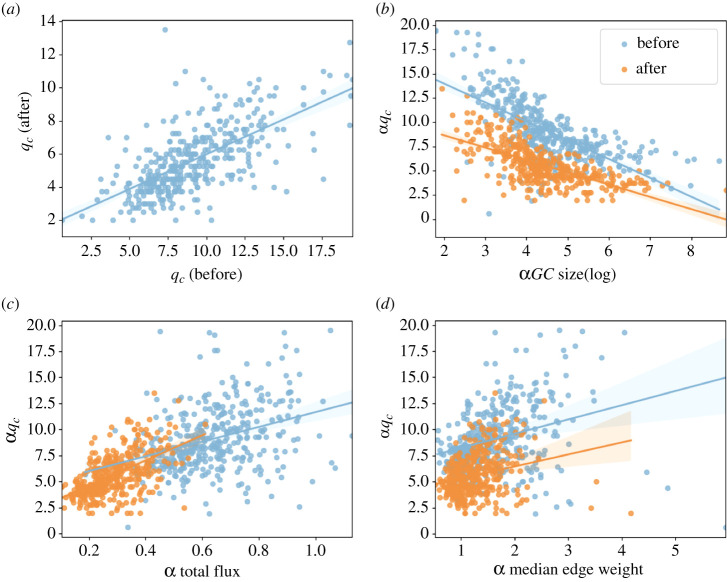
The relationship between $\alpha q_{c}$ and MSA attributes before (29 January to 4 February) and after (29 May to 4 June) national emergency declaration. (*a*) $\alpha q_{c}$ before the national emergency declaration versus $\alpha q_{c}$ after. Panel (*b*–*d*) shows $\alpha q_{c}$ versus the size of αGC at $\alpha q_{c}$, total flux, and median edge weight of each MSA before and after national emergency declaration, respectively. (Online version in colour.)

We compute daily $\alpha q_{c}$s of all MSAs from 1 January to 31 December 2020 and examine these values with MSA attributes. Figure 3*a* shows $\alpha q_{c}$s in different MSAs before and after the national emergency declaration. The values are positively related. Then we explore a few attributes of the MSAs and their correlations with $\alpha q_{c}$ to better understand network structures. Three attributes are tested, and their regression trendlines are shown in figure 3*b*–*d*. Figure 3*b* shows the correlation between $\alpha q_{c}$ and the sizes of the largest components in the MSAs. As $\alpha q_{c}$ increases, the sizes of the largest components at critical points quickly decrease. Comparing the critical points before and after the national emergency declaration, we find that the sizes of the largest components at critical points remain consistent. Yet, $\alpha q_{c}$s during the pandemic are larger than the ones before. Figure 3*c* shows the correlation between $\alpha q_{c}$ and total flux. It is found that the total flux of an MSA increases as $\alpha q_{c}$ increases. Also, the total flux after the national emergency declaration is smaller by comparing points before and after. The change is caused by the decline of local mobility due to the stay-at-home order. Also, median edge weight and $\alpha q_{c}$ are positively correlated (figure 3*d*). There has been no substantial change in the linear correlation magnitude between median edge weight and $\alpha q_{c}$ before and after the national emergency declaration. However, the scatter plot in figure 3*d* suggests a reduction of the median edge weights for αGCs after the declaration with about 50% decrease across most MSAs.

## 6. Inter-MSA percolation

**Figure 4.**
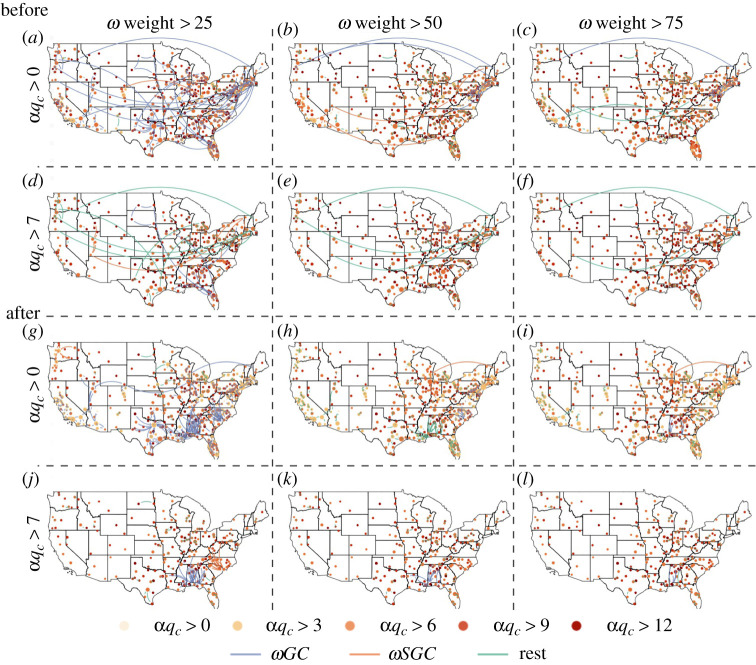
Inter-MSA networks before (i.e. 29 January to 4 February, (*a*–*f*)) and after (i.e. 29 May to 4 June, (*g*–*l*)) the national emergency declaration. The networks are controlled by two parameters: $\alpha q_{c}$ for each MSA, and the edge weights between MSAs. (Online version in colour.)

After analysing the intra-MSA network, we examine the percolation process of the inter-MSA network (i.e. ω-network) in the USA. In figure 4, we control two parameters: $\alpha q_{c}$ for each intra-MSA network and the edge weights between MSAs. As the threshold for weight increases (rows in figure 4), the number of edges decreased, especially for the long-distance links. As the threshold for $\alpha q_{c}$ increases (columns in figure 4), fewer nodes remain and the network becomes sparser. By comparing the corresponding graphs at the same thresholds for edge weight and $\alpha q_{c}$ before and after the national emergency declaration, i.e. figure 4*a*,*g*, we found that $\alpha q_{c}$ for each MSA is smaller in most cases. Also, most long-distance edges in the inter-MSA network before the national emergency declaration disappear in the one after. The changes indicate that while human mobility substantially decreased under the influence of COVID-19, long-distance travels are particularly excluded from people’s travel behaviours. The behaviour changes divide the ω-network into isolated clusters. Also, people are more likely to travel locally under the influence of the pandemic since more edges appear in the local networks, especially in the southeastern region, after the national emergency declaration. While this could indicate strengthened social bonding during the difficult time, the increase could also be a factor contributing to the fast growth of infection cases in these regions.

**Figure 5.**
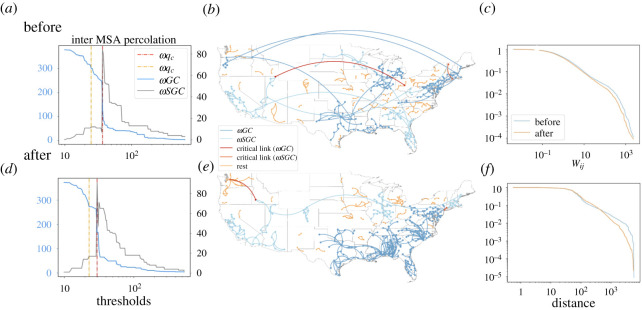
Percolation of inter-MSA mobility network. *a*,*d* show the changes of the sizes of ωGC and ωSGC from 29 January to 4 February 2020 and 29 May to 4 June, respectively. Panel (*b*,*e*) shows the geographical networks when the weight of edges equals $\omega q_{c}$ in the same week. Panel (*c*) shows the complementary cumulative distribution function (CCDF) of normalized edge weight from 29 January to 4 February (blue line) and 29 May to 4 June (red line). (*f*) CCDF of distances of the edges from 29 January 29 to 4 February (blue line) and 29 May to 4 June (red line). (Online version in colour.)

By applying our percolation approach used in the intra-MSA networks, we obtain a similar phase transition pattern for inter-MSA networks. In figure 5*a*, we found that when thresholds reach 4, the size of ωSGC increases abruptly to 80 out of the 378 MSAs. This means nearly $\frac{1}{5}$ of MSAs disconnected from ωGC when the edge weight is 4. At the critical point, the size of ωSGC decreases to less than 100 nodes (figure 5*a*,*b*), and the inter-MSA network loses its strong connectivity to spread COVID-19 effectively.

We observe a similar phase transition during the pandemic (in figure 5*d*). There is an abrupt decrease in the inter-MSA ωGC and an increase in the intra-MSA ωSGC. However, both $\omega q_{c}$ and $\omega q_{c2}$ after the national emergency declaration are smaller than the ones before. The decreases demonstrate people are less likely to travel long-distance under the influence of pandemic and travel restriction.

Figure 5*b* shows the inter-MSA network map at its critical point before the national emergency declaration and figure 5*e* shows one after. By comparing the inter-MSA maps at their critical points, we find several subcomponents in ωGC or ωSGC, including: west region, midwestern region, northeast region, southeast region and south region. The comparison shows that the critical link usually connects these regions. While internal connections within each region remain stable even during the pandemic, cutting off the critical links can effectively disconnect the regions.

The CCDF plot in figure 5*c* shows the difference of distribution edge weight and in figure 5*f* shows the difference of distribution edge distance between two nodes in these two inter-MSA networks. The total numbers of edges of the two networks are the same since there are travellers in any pair of MSAs every day. However, the distribution becomes more heavy-tailed after the national emergency declaration, suggesting more centralized low weight edges and short-distance edges in the ω-networks during the pandemic.

**Figure 6.**
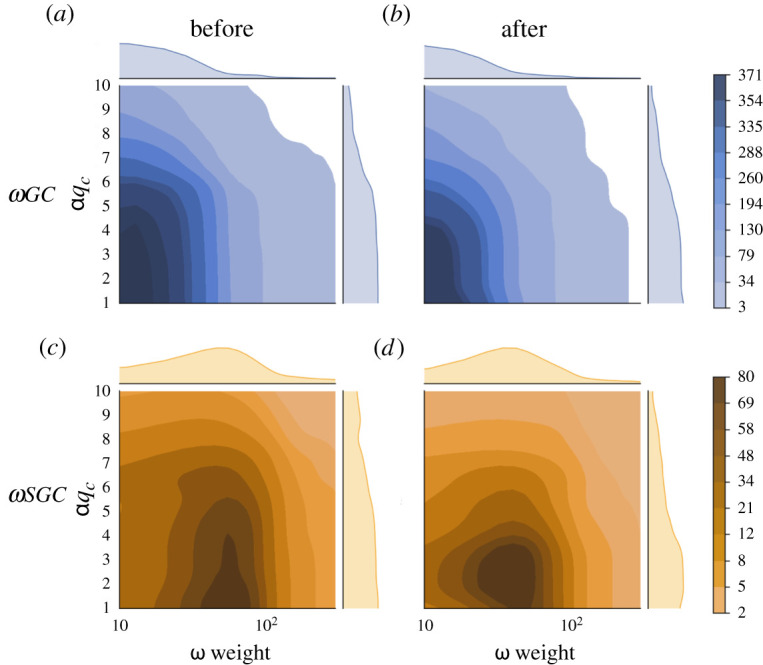
Heat map of $\omega q_{c}$ with different thresholds of $\alpha q_{c}$ and ω weight in percolation of inter-MSA mobility map before (29 January to 4 February) and after (29 May to 4 June) the national emergency declaration. Panel (*a*) shows the heat map of ωGC with $\alpha q_{c}$ and ω weight in the week of 29 January to 4 February 2020. Panel (*b*) shows the heat map of ωGC with $\alpha q_{c}$ and ω weight in the week of 29 May to 4 June 2020. Panel (*c*) shows the heat map of ωSGC with $\alpha q_{c}$ and ω weight in the week of 29 January to 4 February 2020. Panel (*d*) shows the heat map of ωSGC with $\alpha q_{c}$ and ω weight in the week of 29 May to 4 June 2020. (Online version in colour.)

Then we expand our percolation process to two-level thresholds, $\alpha q_{c}$ as each intra-MSA network and ω weight as the normalized weight of edges between MSAs, to take the robustness of different MSAs into consideration. In figure 6, we control these two parameters and create a heat map for both ωGC and ωSGC before and after the national emergency declaration. We observe that before and after the national emergency declaration, both ωGC and ωSGC get smaller as $\alpha q_{c}$ or ω weight get larger. By comparing the same components at different times (ωGC in figure 6*a*,*b* and ωSGC in figure 6*c*,*d*), we can see that both are more centralized in lower $\alpha q_{c}$ and ω weight after the national emergency declaration than before. Thus besides ω weight as we showed, mobility is also more sensitive to $\alpha q_{c}$ after the national emergency declaration.

## 7. Time series of percolation threshold predictability

**Figure 7.**
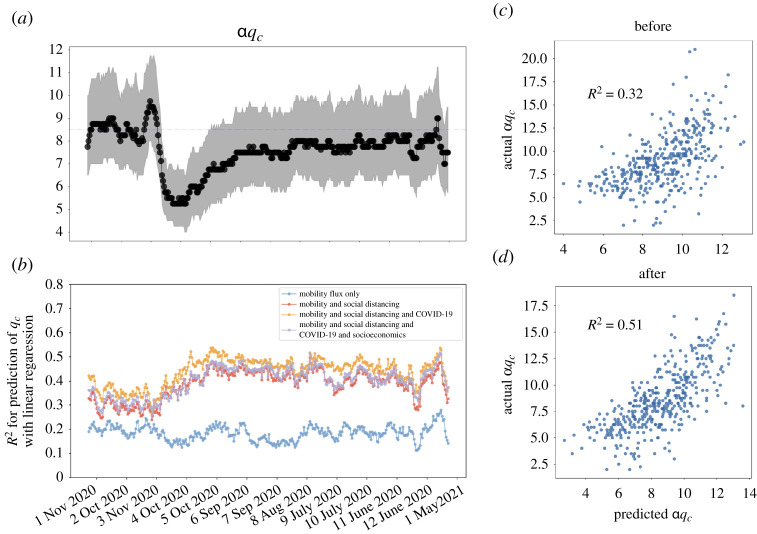
Time series of critical threshold value from 1 January 2020 to 31 December 2020. (*a*) interquartile range of $\alpha q_{c}$ and the line represents the median of $\alpha q_{c}$ in intra-MSA over time. (*b*) Times series of $R^{2}$ for prediction of $\alpha q_{c}$ with linear regression. Scatter plots of predicted $\alpha q_{c}$ with linear regression with respect to the actual value with weekly mean of 29 January to 4 February 2020 (before, *c*) and 6 May to 12 May 2020 (after, *d*). (Online version in colour.)

Next, we investigate the chronological progression of $\alpha q_{c}$ and measure the predictability with various features for all intra-MSA networks. Figure 7*a* captures the time series of descriptive statistics of $\alpha q_{c}$ for each MSA. We find that before the national emergency declaration, the $\alpha q_{c}$ remains stable, with a median value of 8.5. The Interquartile Range (IQR) spans between 7 and 11 with longer tails near the upper quantiles. Right ahead of the declaration, the median value witnessed a sudden increase and reached 10 followed by an abrupt and blunt drop to 5 by the end of March. It is notable that during this sharp decrease, the variance between different MSAs shrank remarkably to less than 1. The reduced variance indicates the universality of perturbation from the emergency declaration across different regions. After the downward trend hitting the lowest point at mid-April, the median value of intra-MSA $\alpha q_{c}$s gradually increased back to 7 around early June and remains stable again.

Figure 7*b* compares the predictive strength of $\alpha q_{c}$ with a different set of features over time. The baseline for the predictors includes the 7-day mobility fluxes with both mobility influx (the total number of travellers from other MSA), outflux (the total number of travellers to other MSA), and intra-flux (the total number of travellers between each pair of census block groups within MSA). We then append different sets of features using the social distancing metrics from SafeGraph, 7-day COVID-19 incremental infection rate, and socio-demographic characteristics. All the attributes, including $\alpha q_{c}$, are obtained as 7-day rolling average except the COVID-19 incremental infection rate, which is computed by 7-day case increase divided by the total population. To mitigate the multicollinearity effect from a high level of correlations between these features, we use principal component analysis (PCA) to ensure the orthogonality before the configuration of the regression models. Given the varying correlation between input features across different days, we apply a uniform threshold of 95% variance to determine the number of principal components being used for prediction. Such a threshold guarantees a minimum of 95% of variance explained across all MSAs on different days. We tested both $R^{2}$ and adjusted $R^{2}$ to consider the change of the number of predictors each day, and the increasing predictive capability over time can be observed for both metrics.

Figure 7*b* shows the $R^{2}$ values with different sets of transformed features. We can see that most combinations of these factors became more predictive of the percolation threshold during the mobility falloff except using mobility flux solely. The overall predictive strength with mobility flux is rather low, with $R^{2}$ values fluctuating around 0.2 over time. With social distancing metrics, the $R^{2}$ values jumped to between 0.3 to 0.35 before mid-March and approached 0.4 around early May. Furthermore, adding the COVID-19 infection rate improves the predictive performance by increasing the $R^{2}$ by 0.1 at most times and exceeded 0.5 around early May. Surprisingly, socio-demographic indicators do not improve the predictive strength and cause even a very minor decrease, which could be due to the lack of variations of these variables on the MSA level. Figure 7*c*,*d* is the scatter plots of prediction versus actual intra-MSA $q_{c}$s, where figure 7*c* shows the week of 29 January to 4 February 2020 when the $R^{2}$ is relatively low (0.32) while figure 7*d* is for 6 May to 12 May 2020 with an $R^{2}$ of 0.51. It is notable that for both cases, the predictions are mostly overestimating the actual outcome. There is also heteroskedasticity present: the error variance changes across different $q_{c}$s. In conclusion, the overall predictive strengths of the percolation threshold with linear methods are moderate. The social distancing behaviour and mobility flow can only partially explain the percolation threshold, while social distancing has played a progressively crucial role since the beginning of the outbreak.

## 8. Discussion and conclusion

In this paper, we study the percolation of the human mobility network that includes over 175 000 of the census block groups in the USA. Two block groups have a connection on a day when users travel between them on that day. If more users travel between them, the weight becomes stronger. However, analysing its percolation is very time-consuming due to the large size, heterogeneous weights, and number of networks. Thus, this paper proposes a novel hierarchical structure of the mobility network to understand human dynamics during the pandemic. In our model, the inter-MSA network (i.e. ω-networks) is composed of 378 MSAs in the USA. Each MSA comprises many census block groups, which is the intra-MSA (i.e. α-networks) level. During the percolation process, we remove the q fraction of the weakest links and measure the giant component of the network because it is easier to break the weak links than the strong links. We find a critical $q_{c}$, and when $q>q_{c}$, the network breaks down abruptly because the large enough second-largest component is disconnected from the giant cluster. We compute daily $q_{c}$ for each intra-MSA and inter-MSA. The analysis allows us to better understand the robustness of each MSA’s connectivity and interactions between MSAs.

In this paper, our analysis of a network’s vulnerability is based on its percolation processes and the transition points. The strength of our percolation analysis is that it breaks the network into isolated clusters with minimum cost. Thus, it can effectively reduce the propagation of COVID-19. However, the method has several limitations. The first limitation is that the percolation process is solely based on the connectivity of a graph [42,43] and thus neglects some essential features of nodes (i.e. ratio of different races, economic status, etc.) and edges (i.e. purpose of people travelling on this route). Taking these features into account can help us better understand the mobility flow and transmission of COVID-19 and develop effective NPI strategies. The second limitation is that mobility behaviours are incredibly complex and dynamic. Thus mobility restriction based on percolation approaches should be considered part of holistic solutions with other NPIs [27]. The third limitation is that results from the percolation process can help contain COVID-19 within a small cluster at the early stage of the pandemic. However, if large-scale community transmission has already started, the method obtained by percolation analysis will be less effective.

The study has three main contributions. The first contribution is the revelation of the hierarchical structures of the temporal mobility network. Given the underlying principle of the cluster forming process in classic percolation theory [34,36], we unveil that spatially hierarchical structure is an essential characteristic of dynamical mobility networks under the impact of COVID-19. Our finding raises the significance of the interaction between different network layers. Thus, the construction of the hierarchical networks enables identifying links that could serve as the critical bridges at different scales, a break of which leads to a disconnection between giant components and second-largest components. Our numerical results provide opportunities to mitigate or prevent macro-transmission with strategic mobility controls.

Secondly, we reveal the universality of phase transition of mobility networks at critical points using percolation theory from statistical physics. Although previous research has demonstrated that the percolation process governs the mobility networks on the county level [31], here we show the percolation process controls the phase transitions in these networks on different levels (i.e. block groups and inter-MSAs) universally. It is supported by the resemblance of functions between the largest components sizes and the percolation threshold across all levels of granularity. The universality demonstrates the possibility of predicting mobility patterns regardless of geographical regions and scales.

Furthermore, the understanding of the association between critical threshold $\alpha q_{c}$ and the key features and characteristics in local MSAs improves our ability to assess and predict the vulnerability of mobility networks. Previous studies show that topological and hierarchical properties of mobility networks and heterogeneous local shocks determine the critical threshold and the emergence of vital adaptive links [31,45]. Here, we quantify the association with various data sources and find that local social distancing metrics are critical factors in predicting the percolation features for time-dependent mobility networks. Surprisingly, despite socio-economic heterogeneities observed from COVID-19’s incidence rate [30], we do not find socio-economic characteristics to be deterministic of the percolation patterns. Future research is needed to help identify other key features and support public health authorities and policymakers to assess the potential local transmission risk.

There are still some limitations in our hierarchical network model, promoting new research directions in the future. First, the data are from device trajectory within a day; thus, some cases are not captured, i.e. people without smartphones, and some are overly counted, i.e. people with multiple devices. Second, we use the partition rule that treats each MSA as a node representing a ‘local network.’ The assumption is that mobility with MSAs is self-containing, i.e. routine travel happens mostly inside MSAs. The drawback of this partition rule is that some rural areas (i.e. vast mid-west region with few MSAs) with low population are not considered in this model, which may cause it to lose some accuracy.

## Data accessibility

Data are private and protected by SafeGraph (https://docs.safegraph.com/docs/social-distancing-metrics). Python code used to process the data and generate the results is made publicly available on the author’s github page (https://github.com/he-h/Covid-Mobility-Network-Analysis).
